# WHO laboratory validation of Xpert^®^ CT/NG and Xpert^®^ TV on the GeneXpert system verifies high performances

**DOI:** 10.1111/apm.12902

**Published:** 2018-11-19

**Authors:** Susanne Jacobsson, Iryna Boiko, Daniel Golparian, Karel Blondeel, James Kiarie, Igor Toskin, Rosanna W. Peeling, Magnus Unemo

**Affiliations:** ^1^ WHO Collaborating Centre for Gonorrhoea and Other Sexually Transmitted Infections National Reference Laboratory for Sexually Transmitted Infections Department of Laboratory Medicine Faculty of Medicine and Health Örebro University Örebro Sweden; ^2^ Clinical Laboratory Department Ternopil Regional Clinical Dermatovenerologic Dispensary Ternopil Ukraine; ^3^ Department of Reproductive Health and Research World Health Organization (WHO) Geneva Switzerland; ^4^ London School of Hygiene and Tropical Medicine (LSHTM) London UK

**Keywords:** GeneXpert, Xpert^®^ CT/NG, Xpert^®^ TV, point of care, *Neisseria gonorrhoeae*, *Chlamydia trachomatis*, *Trichomonas vaginalis*

## Abstract

Effective tests for diagnosis of sexually transmitted infections (STIs), used point of care to inform treatment and management decisions, are urgently needed. We evaluated the analytical sensitivity and specificity of the Xpert^®^ CT/NG and Xpert^®^ TV tests, examining 339 samples spiked with phenotypically and/or genetically diverse strains of *Neisseria gonorrhoeae*,* Chlamydia trachomatis*, and *Trichomonas vaginalis*, and other related species that may cross‐react. The APTIMA Combo 2 test and APTIMA TV test were used as reference tests. The analytical sensitivity for all three agents in the Xpert^®^ CT/NG and Xpert^®^ TV tests was ≤10^2^ genome equivalents/reaction. The analytical specificity of both tests was high. False‐positive results were identified in the Xpert^®^ TV test when challenging with high concentrations of *Trichomonas tenax*,* Trichomonas gallinae, Trichomonas stableri*, and *Trichomonas aotus*. However, the clinical relevance of these cross‐reactions can likely be neglected, because these species have not been identified in urogenital samples from humans. In conclusion, the analytical sensitivity and specificity of the user‐friendly Xpert^®^ CT/NG and Xpert^®^ TV tests on the GeneXpert system were high. The results support the use of specimens from also extra‐genital sites, for example, pharynx and rectum. However, appropriate clinical validations are additionally required.

The World Health Organization (WHO) estimated in 2012 that *Trichomonas vaginalis* (TV) causes ~143 million cases of trichomoniasis in men and women globally per year, *Chlamydia trachomatis* (CT) ~131 million cases of chlamydia, and *Neisseria gonorrhoeae* (NG) ~78 million cases of gonorrhea. If these frequently asymptomatic STI cases are not detected and treated, they might result in severe complications and sequelae 1. In many settings internationally, laboratory detection of these STIs is suboptimal or totally absent.

Traditionally, laboratory diagnosis of these infections has mostly relied on microscopy and/or culture 2, 3. During the last two decade(s), nucleic acid amplification tests (NAATs) have become the recommended diagnostic methods, due to superior performance characteristics 3. However, the NAATs are technically challenging, laboratory based, and too expensive for less‐resourced settings 1. Point‐of‐care tests (POCTs), providing results at the time of the patient visit, would be exceedingly valuable in particularly less‐resourced settings but also more‐resourced settings. POCTs enable early detection of the specific STI agents and guide treatment, forestalling development of sequelae and adverse events, interrupting onward transmission, and offering opportunities for counseling and partner notification. An ideal POCT should fulfill the WHO ASSURED criteria, namely be affordable, sensitive, specific, user‐friendly, rapid and robust, equipment‐free, and deliverable to the end user 4. Simple, rapid, and equipment‐free POCTs for the diagnosis of CT, NG, and TV infections are commercially available; however, in general, these POCTs have a suboptimal sensitivity compared to NAATs, especially when used with noninvasive specimens such as vaginal swabs and urine 5, 6, 7, 8. Emerging technologies promise major advancements in the field of STI POCTs in the coming years. It is crucial to evaluate the performance characteristics of these new tests and their acceptability to patients and health‐care providers. The WHO facilitates access to adequate STI POCTs within national STI programs through comprehensive validation of new promising STI POCTs. This WHO validation is performed in two steps: (i) a comprehensive analytical laboratory validation and (ii) subsequent clinical validation(s) of POCTs that show(s) appropriate results in the laboratory validation.

The Xpert^®^ CT/NG and Xpert^®^ TV tests for the GeneXpert system are NAAT‐based tests that can be used at point of care. The GeneXpert system is fully automated, cartridge‐based, and integrates sample processing, cell lysis, purification, nucleic acid amplification, and detection. The Xpert^®^ TV detects one TV target (TV) and the Xpert^®^ CT/NG simultaneously detects CT and NG by amplifying one chromosomal target (CT1) for CT and two separate chromosomal targets (NG2 and NG4) for NG. Both NG targets need to be detected for the Xpert^®^ CT/NG assay to return a positive NG result.

Our aim was to evaluate the analytical sensitivity and specificity of the Xpert^®^ CT/NG and Xpert^®^ TV tests on the GeneXpert system and, in particular, challenge the specificity with various non‐NG Neisseria, non‐CT Chlamydia, and non‐TV Trichomonas species as well as other closely related bacteria and agents associated with vaginal discharge, to strictly evaluate if particularly the Xpert^®^ CT/NG assay can be used also for specimens from extra‐genital sites such as pharynx and rectum.

## Materials and Methods

The evaluation panel consisted of 339 samples mostly spiked with geographically and temporally (1971–2016) diverse isolates of NG, CT, and TV as well as non‐NG Neisseria, Moraxella, Trichomonas, Chlamydia, Candida species, or bacterial vaginosis specimens. The panel was assembled to include phenotypically and/or genetically diverse strains. For NG, the well‐characterized and geographically, temporally, and genetically diverse WHO reference strains 9, which include *porA* and *cppB* mutants that have previously escaped diagnostics using other NAATs, were included. Due to the high genetic heterogeneity of non‐NG Neisseria species and problems with cross‐reactivity in several gonococcal NAATs 10, a high number of isolates (n = 251) representing 15 different non‐NG Neisseria species were tested to substantially challenge the specificity of the Xpert^®^ CT/NG. For CT, all main genotypes, including *Lymphogranuloma venereum* (LGV), and mutants, such as the Swedish new variant of CT (nvCT), were represented.

To investigate analytical sensitivity, 10 spiked samples each of NG, CT, and TV with known genome equivalents (GEQs) concentrations (10^2^–10^6^ GEQs per reaction) based on quantitative PCR using the *C. trachomatis*/*N. gonorrhoeae*/*M. genitalium*/*T. vaginalis*‐multiprime‐FRT PCR kit (Ecoli s.r.o**.**, Bratislava, Slovak Republic) on the Rotor‐Gene Q instrument (Qiagen, Hilden, Germany) and quantified Amplirun DNA controls (Vircell, Granada, Spain) were analyzed.

To substantially challenge the analytical specificity of the Xpert^®^ TV test, 10 samples including four different non‐TV Trichomonas species were tested in two concentrations (~2.5 × 10^6^ GEQs/test and ~1.25 × 10^7^ GEQs/test, respectively). DNA was purified using MagNA Pure Compact Nucleic Acid Isolation Kit I (Roche Diagnostics GmbH, Mannheim, Germany), added into a transport reagent tube and then transferred to the reaction cartridge, according to the manufacturer's instructions (Cepheid, Sunnyvale, CA, USA). To challenge the specificity of the Xpert^®^ CT/NG assay, 10 samples, including four different non‐CT Chlamydial species in two concentrations (~2.2 × 10^6^ GEQs/test and ~1.1 × 10^7^ GEQs/test, respectively), were tested in the same way as for Trichomonas. Furthermore, 254 samples, including 15 different non‐NG Neisseria and three Moraxella species, were tested in high concentration as previously described 10; two colonies were added into a transport reagent tube and transferred to the reaction cartridge (corresponding to ~2 × 10^7^ colony‐forming units (CFUs)/test), according to the manufacturer's instructions (Cepheid, Sunnyvale, California, USA). In addition, 25 samples of other causes of vaginal discharge; five each of swab samples positive for NG, CT, TV, bacterial vaginosis, and Candida species, as well as 10 negative samples consisting of buffer only were examined. Finally, to mimic human sample conditions, 10 ng/μL of K562 Human DNA high molecular weight (Promega, Madison, WI USA) was added to each reaction/cartridge. If any cross‐reactivity was identified, the false‐positive sample was diluted to evaluate the clinical relevance of the cross‐reaction.

The APTIMA Combo 2 test and APTIMA TV test (Hologic, Marlborough, MA, USA) were used as reference tests and performed according to the manufacturer's instructions (Table 1).

**Table 1:** Positivity rate and comparison between GeneXpert and APTIMA assays for the detection of *Neisseria gonorrhoeae* (NG), *Chlamydia trachomatis* (CT), and *Trichomonas vaginalis* (TV) in 339 mostly spiked samples

Bacterial species tested	No. of isolates tested	Positive Xpert^®^ CT/NG		Positive Xpert^®^ TV	Positive APTIMA CT/NG	Positive APTIMA TV
		CT	NG			
*N. gonorrhoeae* WHO F	1	0	1	0	1	0
*N. gonorrhoeae* WHO G	1	0	1	0	1	0
*N. gonorrhoeae* WHO L	1	0	1	0	1	0
*N. gonorrhoeae* WHO M	1	0	1	0	1	0
*N. gonorrhoeae* WHO U	1	0	1	0	1	0
*N. gonorrhoeae* WHO V	1	0	1	0	1	0
*N. gonorrhoeae* WHO W	1	0	1	0	1	0
*N. gonorrhoeae* WHO X	1	0	1	0	1	0
*N. gonorrhoeae* WHO Y	1	0	1	0	1	0
*N. gonorrhoeae* WHO Z	1	0	1	0	1	0
*N. gonorrhoeae* positive swabs	5	0	5	0	5	0
All *N. gonorrhoeae*	15	0	15	0	15	0
*N. animalis*	1	0	0	0	0	0
*N. bergeri*	1	0	0^b^	0	0	0
*N. cinerea*	9	0	0	0	0	0
*N. elongata*	3	0	0	0	0	0
*N. flava*	1	0	0	0	0	0
*N. flavescens*	90	0	0	0	0	0
*N. lactamica*	12	0	0	0	0	0
*N. macacae*	17	0	0	0	0	0
*N. mucosa*	18	0	0	0	0	0
*N. oralis*	1	0	0^b^	0	0	0
*N. perflava*	62	0	0	0	0	0
*N. sicca*	9	0	0	0	0	0
*N. subflava*	6	0	0	0	0	0
*N. gonorrhoeae subspecies kochii*	4	0	0	0	0	0
*N. meningitidis* a	17	0	0	0	0	0
*M. catarrhalis*	1	0	0	0	0	0
*M. nonliquefaciens*	1	0	0	0	0	0
*M. osloensis*	1	0	0	0	0	0
All non‐NG Neisseria or Moraxella species	254	0	0	0	0	0
*C. trachomatis* Ba	1	1	0	0	1	0
*C. trachomatis* D	1	1	0	0	1	0
*C. trachomatis* E	1	1	0	0	1	0
*C. trachomatis* F	1	1	0	0	1	0
*C. trachomatis* G	1	1	0	0	1	0
*C. trachomatis* H	1	1	0	0	1	0
*C. trachomatis* J	1	1	0	0	1	0
*C. trachomatis* K	1	1	0	0	1	0
*C. trachomatis* nvCT	1	1	0	0	1	0
*C. trachomatis* LGV (L2b)	1	1	0	0	1	0
*C. trachomatis* positive swabs	5	5	0	0	5	0
All *C. trachomatis*	15	15	0	0	15	0
*C. suis* (ATCC VR‐1474)	2	0	0	0	0	0
*C. muridarum* (ATCC VR‐123)	2	0	0	0	0	0
*Chl. pneumoniae* (ATCC VR‐2282, MBC011)	4	0	0	0	0	0
*Chl. psittaci* (MBC013)	2	0	0	0	0	0
All non‐CT Chlamydia species	10	0	0	0	0	0
*T. vaginalis* (incl. ATCC 30001, 50140)	10	0	0	10	0	10
*T. vaginalis* positive swabs	5	0	0	5	0	5
All *T. vaginalis*	15	0	0	15	0	15
*T. aotus* (ATCC 50649)	2	0	0	1	0	0
*T. gallinae* (ATCC 30002, 30230)	4	0	0	4	0	0
*T. stableri* (ATCC PRA‐412)	2	0	0	2	0	0
*T. tenax* (ATCC30207)	2	0	0	2	0	0
All non‐TV Trichomonas species	10	0	0	9	0	0
Bacterial vaginosis positive swabs	5	0	0	0	0	0
Candida ssp. positive swabs	5	0	0	0	0	0
Negative samples	10	0	0	0	0	0

WHO, World Health Organization; LGV, lymphogranuloma venereum.

a Representing all major meningococcal clones spreading worldwide, including serogroups A, B, C, E, W, X, Y, and Z.

b Isolates displaying positivity in the NG4 target in the Xpert^®^ CT/NG test.

## Results

All 10 WHO NG reference strains 9 were positive for both NG targets (NG2 and NG4) in the Xpert^®^ CT/NG assay and detected at ≤10^2^ GEQs per reaction (Table 1). All 254 non‐NG Neisseria species and Moraxella species were negative in the Xpert^®^ CT/NG assay. One (NG4) of the two NG‐targets were repeatedly detected in one *N. oralis* isolate and one *N. bergeri* isolate; since both targets need to be detected for a positive result, they were reported as negative for NG by the GeneXpert^®^ CT/NG assay software. By further dilution of the *N. oralis* and *N. bergeri* isolates (1/10 000; to ~2 × 10^7^ CFUs/test), both tested negative also for the NG4 target.

All 10 CT samples were detected at ≤10^2^ GEQs per reaction in the Xpert^®^ CT/NG assay. No false‐positive samples were detected among the non‐CT Chlamydia species tested, *C. suis* (ATCC VR‐1474), *C. muridarum* (ATCC VR‐123), *Chl. pneumoniae* (ATCC VR‐2282, MBC011), and *Chl. psittaci* (MBC013) (Table 1).

All 10 TV samples, including the two TV ATCC strains 30001 and 50140, were detected at ≤10^2^ GEQs per reaction in the Xpert^®^ TV assay. In the ~1.25 × 10^7^ GEQs/test concentration, all four non‐TV Trichomonas species examined, that is, *T. tenax* (ATCC 30207), *T. gallinae* (ATCC 30002), *T. gallinae* (ATCC 30230), *T. stableri* (ATCC PRA‐412), and *T. aotus* (ATCC 50649) were also positive. In the ~2.5 × 10^6^ GEQs/test concentration, all except *T. aotus* (ATCC 50649) remained positive.

No cross‐reactivity was detected in the Xpert^®^ CT/NG test or Xpert^®^ TV test when testing samples of other causes of vaginal discharge such as bacterial vaginosis and Candida species.

The reference tests (APTIMA Combo 2 and APTIMA TV) showed 100% sensitivity and specificity for all tested samples (Table 1).

## Discussion

In the present study, the Xpert^®^ CT/NG test on the GeneXpert system displayed a very high analytical sensitivity and specificity when substantially challenged with a wide range of genetically diverse NG and CT strains and various non‐NG Neisseria (including 251 isolates of 15 different non‐NG Neisseria species) and non‐CT Chlamydia species, as well as other closely related bacteria and agents associated with vaginal discharge. Accordingly, we could verify the high sensitivity and specificity of the Xpert^®^ CT/NG test, which has been indicated in previous studies 10, 11, 12, 13. Our findings further support the use of the Xpert^®^ CT/NG test for the detection of CT and NG in also specimens from extra‐genital sites such as pharynx and rectum. We could also verify the high sensitivity and specificity of the Xpert^®^ TV test, which has been indicated in two previous studies 14, 15. However, we also show that several non‐TV Trichomonas species, in high concentrations, were also positive in the Xpert^®^ TV test. Cross‐reaction with *T. tenax*, a protozoan commonly found in the oral cavity of humans, dogs, and cats, has been identified earlier, including by the manufacturer at levels above 1 × 10^2^ cells/mL 16. We identified for the first time cross‐reaction also with the avian protozoa *T. gallinae* and *T. stableri* 17, both mainly found in the oral–nasal cavity and infecting the respiratory or upper digestive tract of birds, as well as *T. aotus* 18, mainly found in the intestines giving gastroenteritis in monkeys. Nevertheless, the clinical relevance of these cross‐reactions with non‐TV Trichomonas species can likely be neglected because, to our best knowledge, these species have not been identified in urogenital samples from humans. Nevertheless, additional studies addressing this issue might be valuable.

The Xpert^®^ CT/NG and Xpert^®^ TV tests on the GeneXpert system fulfill several of the WHO ASSURED criteria 4. However, the tests do not fulfill the criteria of being sufficiently inexpensive, rapid, and equipment‐free. Nevertheless, despite the relatively high cost per test, the significant benefits associated with the access to POC diagnostics (in some settings, results can be provided at the time of the patient visit), specific detection and treatment of etiological agent of STIs (reducing over‐ or undertreatment), reduced time to treat, and opportunities for counseling and contact notification need to be taken into account in cost‐effectiveness analysis. The Xpert^®^ CT/NG test and Xpert^®^ TV test on the GeneXpert system is also relatively rapid and produces results from the primary sample in 1.5 and 1 h, respectively. Furthermore, the GeneXpert system has been considered as suitable for use in remote health‐care settings, low‐resource settings, and settings with limited laboratory infrastructure 19, which otherwise frequently have to rely on substantially delayed results from, for example, a central laboratory. In a previous study 20, it was concluded that the commercially available NAAT POCTs do not produce results quickly enough to be provided before most patients leave the clinic. However, the total time to treatment per 100 infected patients was estimated to decrease by 204–208 days for CT infection and 164–172 days for gonorrhea based on the estimated reduction in time to obtain results to one day from the current median of three to four working days but this at the expense of a few false‐positive and false‐negative results 20. The reduced time to treatment may have significant implications for prevention of further disease transmission.

In conclusion, the analytical sensitivity and specificity of the Xpert^®^ CT/NG and Xpert^®^ TV tests on the GeneXpert system were high. The high analytical specificity of the Xpert^®^ CT/NG assay supports also the use of specimens from extra‐genital sites such as pharynx and rectum; however, appropriate clinical validations are required and the WHO has most recently initiated a large international clinical validation, including also specimens from pharynx and rectum. These tests used on the GeneXpert system are also user‐friendly (for laboratory staff but also other health‐care professionals) and relatively rapid. Nevertheless, sensitive and specific STI diagnostic POCTs that are more rapid and cheaper would be valuable and the development and validation of such assays are key priorities for the management and control of curable STIs in the future.

We are grateful to Cepheid for providing Xpert^®^ CT/NG and Xpert^®^ TV tests. The present work was supported by the Department of Reproductive Health and Research, World Health Organization (WHO), Geneva, Switzerland; Örebro County Council Research Committee and the Foundation for Medical Research at Örebro University Hospital, Örebro, Sweden.
